# Case Report: Hemoglobin titusville: a specific case of cyanosis

**DOI:** 10.3389/fcvm.2025.1537704

**Published:** 2025-06-17

**Authors:** Meng Zhang, Kai Wang, Xinyi Xu, Wei Ji, Ying Guo, Wei Gao, Tingliang Liu

**Affiliations:** Department of Cardiology, Shanghai Children's Medical Center, School of Medicine, Shanghai Jiao Tong University, Shanghai, China

**Keywords:** hemoglobinopathy, hemoglobin titusville, hypoxemia, cyanosis, cardiac catheterization

## Abstract

Hemoglobin Titusville is a rare low-oxygen-affinity hemoglobinopathy characterized by normal arterial partial pressure of oxygen but low oxygen saturation. In this study, we describe a case of hemoglobinopathy presenting as cyanosis. A 12-year-old female patient was admitted to the hospital with cyanosis and 87% blood oxygen saturation. Overall, the patient was healthy and preserved exercise tolerance. Upon admission, her arterial partial pressure of oxygen was 121 mmHg, and her blood oxygen saturation was 89.7%. First, the common causes were excluded, after which cardiovascular catheterization was performed. This detected no shunt between the systemic and pulmonary circulation. Finally, whole-exon sequencing confirmed a heterozygous mutation of the *HBA1* gene (g.227115G>A). In this study, we review the entire course of this case, providing scientific insight into this disease and a differential diagnosis of cyanosis.

## Introduction

Hemoglobin (Hb), an essential protein in the human body, transports oxygen to tissues throughout the body. Diseases affecting Hb synthesis and function are very common, with 269 million carriers worldwide (1, 2). Low-oxygen-affinity Hb variants can be detected through arterial blood gas analysis when blood arterial oxygen saturation (SaO_2_) is low compared with normal arterial partial pressure of oxygen (PaO_2_) (3). In such cases, the biochemical properties of the variant are assumed to induce the reduction in SaO_2_. This article describes a case of a diagnosis of Hb Titusville, a rare Hb-related disease. Owing to a mutation in the gene-encoding globin, the affinity of Hb for oxygen and its oxygen-carrying capacity of Hb are decreased, with patients developing hypoxemia (4).

## Case report

A 12-year-old girl had cyanosis of the lips since early childhood. However, because of her good growth and development, no prior medical evaluation was pursued. She was admitted to a local hospital for mycoplasma pneumonia, with a pre-pneumonia SpO_2_ of 87% this time. Nonetheless, after the pneumonia was treated, transcutaneous oxygen saturation SpO_2_ was still 87%. In local hospital, she was only treated with antibiotics and doctors considered pulmonary arteriovenous fistula, which required further diagnosis. At the time of admission to our hospital, pulmonary infection resolved fully. She did not experience chest tightness, shortness of breath, chest pain, or other symptoms after strenuous activities.

**Figure 1:**
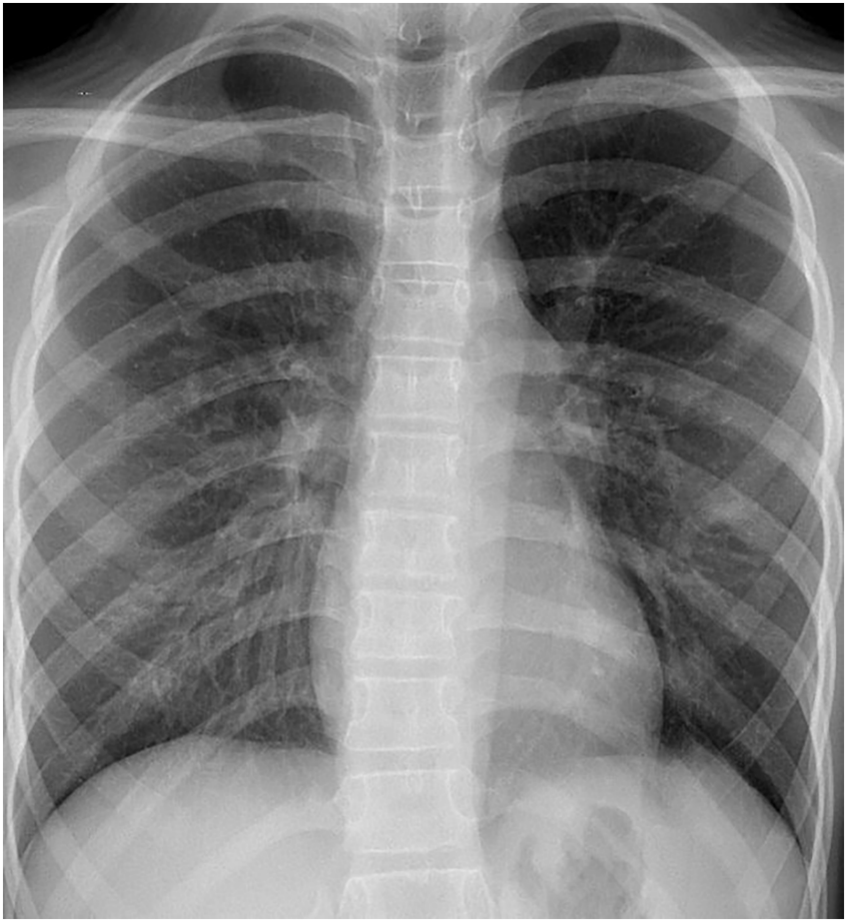
Chest plain radiograph of the child at the time of admission; no basis for pulmonary arteriovenous fistula was found.

**Figure 2:**
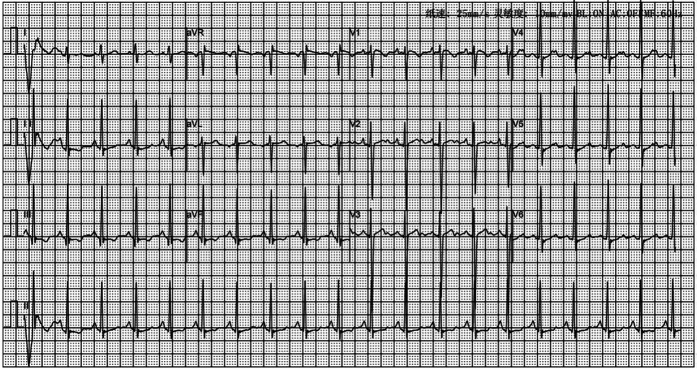
Electrocardiogram of the child at the time of admission; no abnormality.

A post-admission physical examination revealed cyanosis of the mouth and lips. Routine physical examinations of the nervous system, heart, lungs, and abdomen indicated no obvious cyanosis of the limbs, no noticeable abnormalities, no clubbing of the fingers or limbs, and no murmurs on cardiopulmonary auscultation. Upon admission, under room air, the patient's transcutaneous oxygen saturation was between 87% and 89%, measured using different pulse oximetry devices. In addition, under room air, results of blood gas analysis revealed that PaO_2_ was 121 mmHg, with 89.7% oxygen saturation. There were no abnormalities in routine laboratory tests, and the results are shown in Table 1. Chest radiographs and 12-lead EKGs are normal (as Figures 1, 2).

**Table 1:** Routine laboratory findings.

Laboratory test	Results	reference range
Leucocyte	6.6 × 10^9 ^/L	4–10 × 10^9 ^/L
Blood platelet	193 × 10^9 ^/L;	100–300 × 10^9 ^/L
Hb	126.0 g/L	120–160 g/L
Red blood cell	4.37 × 10^12 ^/L	4.0–5.5 × 10^12 ^/L
Erythrocyte specific volume assay	38.3%	38%–50%
Mean erythrocyte volume	87.6 fL	80–100 fL
Mean erythrocyte hemoglobin content	28.8 pg	27–33 pg
Mean erythrocyte hemoglobin concentration	329 g/L	320–360 g/L
Prothrombin time	13.3 s	9.8–13 s
Partial thromboplastin time	35.6 s	22.5–34 s
Fibrin monomer	4.60 g/L	2–4.5 g/L
Alkaline phosphatase	197 U/L	53–128 U/L
Aspartate aminotransferase	19 U/L	0–30 U/L
Alanine aminotransferase	12 U/L	0–40 U/L
Creatinine	44 μmol/L	50–120 μmol/L
Urea	2.2 mmol/L	2.0–6.3 mmol/L
Lactate	1.87 mmol/L	0.6–2.2 mmol/L
Blood ammonia	27 μmol/L	9–30 μmol/L
Total bilirubin	10 umol/L	3.4–17.1 umol/L
Gamma-glutamyl transferase	12 U/L	0–50 U/L
CKMB	<0.50 ng/L	<0.50 ng/L
Troponin I	0.016 μg/L	<0.2 µg/L
Sodium	137.6 mmol/L	135–145 mmol/L
Potassium	3.88 mmol/L	3.5–5.5 mmol/L
Carbon dioxide binding	21.0 mmol/L	20–29 mmol/L
FEV1	94%	Normal
FEV1/FEV	93%	Normal
DLCO	85%	

Based on the characteristics of cyanosis, which was uniformly distributed throughout the body in the skin, mucous membranes, and warm skin, we first considered the type of cyanosis to be central cyanosis. We performed a series of examinations and tests according to the diagnostic and therapeutic concepts of central cyanosis. We focused on the respiratory system, circulatory system, vascular diseases, and hematologic diseases. We also inquired about an overdose of relevant medications and a history of relevant special foods, which were all negative. Her head MRI screening was also normal.

Concerning the respiratory system, the investigation was focused on asthma and other obstructive ventilation dysfunction disorders, as well as disorders of the ventilation–perfusion ratio such as pulmonary embolism and severe pneumonia. A series of examinations including chest HRCT, pulmonary ventilation function test and pulmonary carbon monoxide diffusion function were obtained. All results were negative. DLCO accounted for 85% of the expected value in this children. The high-resolution CT enhancement was also negative, ruling out prolonged hypoxemia induced by complex respiratory disease.Cardiac catheterization was also performed to assess the development of pulmonary vessels and to rule out pulmonary embolism.

**Figure 3:**
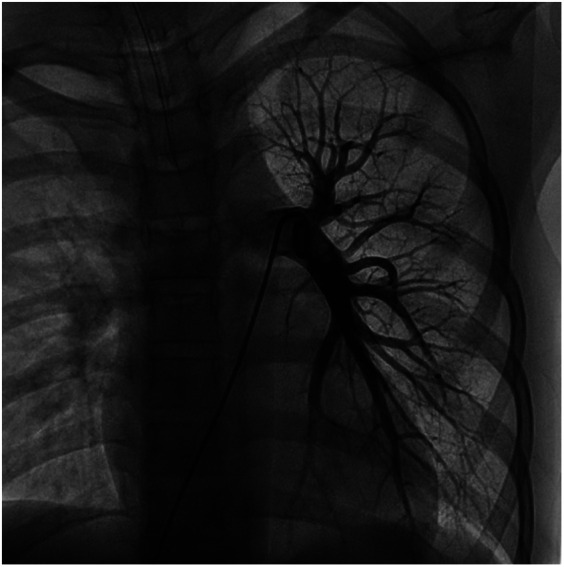
Frontal and lateral views of the child's left pulmonary arteriogram.

**Figure 4:**
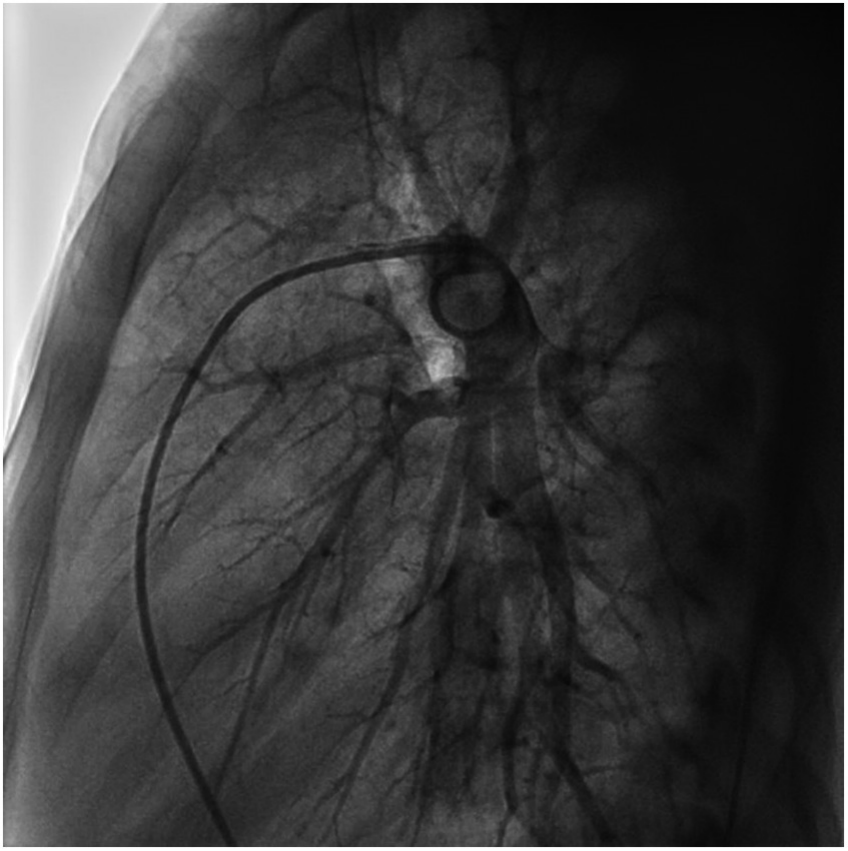
Frontal and lateral views of the child's left pulmonary arteriogram.

**Figure 5:**
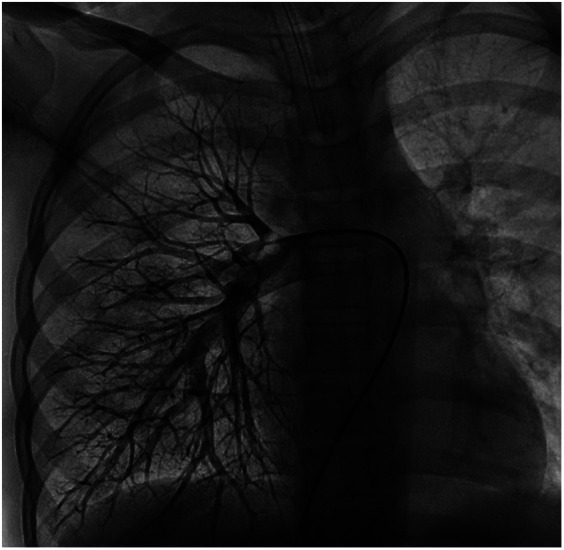
Frontal views of the child's right pulmonary arteriogram. No pulmonary arteriovenous fistula was found.

**Figure 6:**
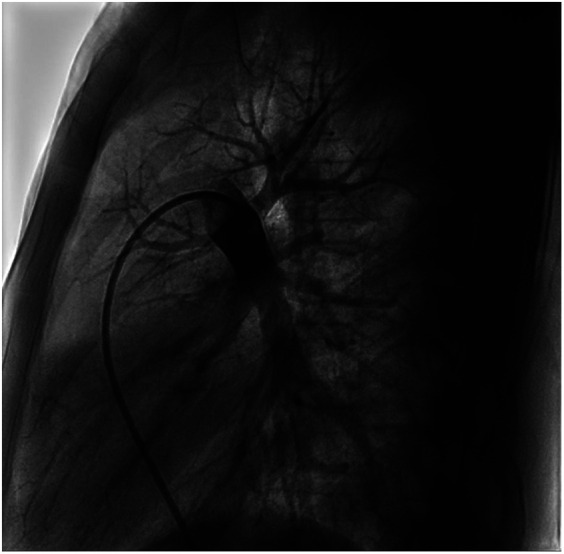
Frontal views of the child's right pulmonary arteriogram. No pulmonary arteriovenous fistula was found.

We performed a detailed evaluation of the cardiovascular system, including tests for right-to-left diverged heart disease, pulmonary arteriovenous fistula, severe valve regurgitation, and pulmonary hypertension.After conducting detailed echocardiography and cardiac CTA, we performed cardiac catheterization. The results of cardiac catheterization were negative. Cardiac catheterization findings are shown in Figures 3–6. We were unable to find a shunt at the atrial, ventricular, or aortic levels. The child's echocardiogram and cardiac macrovascular CT results were normal, ruling out complex congenital heart disease such as pulmonary atresia.

After we ruled out diseases related to the cardiopulmonary system, it was considered most likely that the cyanosis was caused by a rare disease of the hematologic system. We first considered hemoglobin disorders such as methemoglobinemia (MetHb) and conducted methylene blue tests. The disease of MetHb is characterized by the oxidation of ferrous iron in hemoglobin to trivalent iron and the loss of oxygen-carrying function. Congenital MetHb is caused by NADH-methemoglobin reductase deficiency and is inherited by autosomal recessive inheritance. We performed the sublethal blue test for this purpose; sublethal blue reduces the trivalent high iron in hemoglobin back to divalent ferrous iron and restores the oxygen-carrying capacity. However, in this case, there was no improvement in the patient's blood oxygen content after the administration of a methylene blue injection and observation for 1 h. After we collected DNA for whole-exon sequencing, the patient was discharged from the hospital.

**Figure 7:**
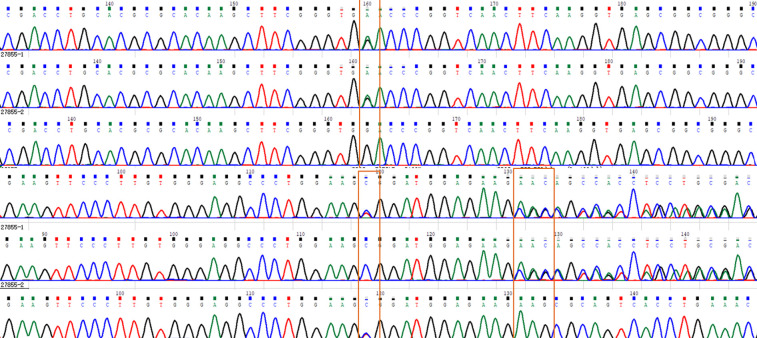
The whole-exon gene sequencing of the patient is presented above, indicating the HBA1 heterozygous mutation c.283G>A,p.D95N.

One month later, the patient's genetic report indicated a heterozygous mutation in the *HBA1* gene, which was carried in the paternal line, located in Chr16 (GRCh37): g.227115G, C.283G>A, P.AS95ASN, (as Figure 7) which could lead to Titusville-type hemoglobinopathy, the clinical manifestation of which was low-affinity hemoglobinopathy. According to the mutation classification standards of the American College of Medical Genetics and Genomics, this mutation was “possibly pathogenic.” Finally, we found that the child's father, who was 44 years old, was also cyanotic and had a transcutaneous oxygen saturation of 88% but no obvious complaints of discomfort.

## Discussion

Hb Titusville is a rare disease, clinically asymptomatic in most cases. Most patients with Hb Titusville display no obvious symptoms or are inadvertently found to have hypoxemia due to other conditions such as asthma, pneumonia, and routine surgery. After the disease is diagnosed, an investigation of the patient's family reveals that they also have the disease. This was first reported in 1975 when Schneider et al. described for the first time the case of a 3-year-old African–American female patient with no clinical symptoms whom researchers found during mass screening for abnormal hemoglobinopathy (1). Mina et al. (2) and Ruetsch et al. (4) provided a detailed overview of the existing literature on Hb Titusville. To date, a total of 14 cases of this disease have been reported, with more than 20 patients in total, with the onset age ranging from newborn infants to 61-year-old patients with cerebral infarction, and these patients exhibit no significant difference in gender and race (5, 6).

Hb is the tetrameric protein for which the coding genes are grouped in two separate globin gene cluster families on different locations in the genome. Hemoglobinopathies, the genetic diseases related to Hb synthesis, constitute the most common monogenic disorders worldwide (7). The genetic cause of this group of diseases are DNA variants in or near the globin genes, coding for the globin chains of the tetrameric haemoglobin protein (7). The crucial function of Hb is carrying oxygen throughout the body. In addition to external factors such as pH value, 2,3-DPG concentration, and temperature, mutation cooperativity in globin-encoding genes that produce new mutant proteins, such as the Hb Titusville protein, greatly affect the oxygen-carrying capacity of Hb. The crux of the problem is that low-affinity Hb does not bind oxygen properly during blood-gas exchange in the lung, so the blood gas analysis reveals that SaO_2_ remains consistently low even if PaO_2_ is normal. Whereas in the tissues, the low affinity Hb will increase delivery oxygen to tissues because it will release oxygen at a higher tissue PaO2 level.

The characteristics of the present case are similar to those reported in the aforementioned studies (5, 8): with no obvious clinical manifestations, persistent hypoxemia still appeared after the pneumonia was cured, but the patient did not experience any discomfort. Following admission, a series of diagnostic tests were conducted, with the disease finally being confirmed by genetic testing.

The principal method of diagnosis is to analyze the Hb of suspected patients via high-performance liquid chromatography (HPLC) or capillary electrophoresis to find abnormal Hb, and finally, the diagnosis is confirmed by whole-exon sequencing (1, 9). In the diagnosis of this case, if we had conducted HPLC earlier, it would have been possible to detect the clues of the disease earlier. Now, close to 900 human variant hemoglobins are known, and new ones are still being discovered (10). Most variant hemoglobins do not cause disease, but some cause hemolytic anemia such as sickle hemoglobin (Hb S) or its instability (Hb Hammersmith). Others cause methemoglobinemia and cyanosis (Hb M-Boston) or polycythemia due to their increased oxygen affinity (Hb Ypsilanti). Other variant hemoglobins have decreased oxygen affinity that usually do not cause clinical symptoms. Hb Titusville is caused by the mutation of codon 94 of the globin α1 chain (GAC>AAC), which leads to the mutation of the encoded amino acid from aspartic acid to asparagine (9). The corresponding protein isomer of the mutation is called Hb Titusville, which is characterized by a low affinity for oxygen. Aspartic acid encoded by codon 94 plays a significant role in the spatial configuration of the globin α chain. For the six known mutations, aspartic acid is replaced by point mutations, such as Hb Bassett, where alanine replaces aspartic acid, and Hb Capa, where glycine replaces aspartic acid. In Hb Roanne, glutamic acid replaces aspartic acid; in Hb Setif, tyrosine replaces aspartic acid; and in Hb Sunshine Seth, histidine replaces aspartic acid (11). All of these mutations lead to decreased Hb affinity for oxygen, decreased cooperativity, and hypoxemia in patients.

The differential diagnosis of the cyanosis and hypoxemia as the primary clinical manifestations is complex, and the diagnosis and treatment ideas should be extensive and comprehensive, particularly for cardiovascular, lung, and hematologic diseases as the primary considerations. The patient was initially admitted to the hospital due to concerns about a “pulmonary arteriovenous fistula”. Scaravilli V et al. (3) reported the physiological characteristics of patients with Hb Titusville by measuring the characteristics of the present case, such as the continuous mixture of intravenous oxygen saturation (SVO_2_), cardiac output, oxygen delivery (DO_2_), oxygen consumption (VO_2_), and the oxygen extraction ratio (ERO_2_). The oxygen dissociation curve of Hb Titusville was described, and the highest P50 points on the curve and the lowest cooperativity of the mutant protein (Hill coefficient of 1.45 vs. 2.27) were confirmed. It was found that in these patients, hypoxia was offset not by an increase in oxygen uptake but by an increase in cardiac output.

Cyanosis directly reflects the concentration of deoxyhemoglobin in the patient's blood. Its causes are complex and involve various systems of the human body. This case provides a scientific diagnosis idea and clinical pathway for the diagnosis and treatment of rare cyanosis diseases, and has good clinical guiding significance.

## Data Availability

The datasets presented in this study can be found in online repositories. The names of the repository/repositories and accession number(s) can be found in the article/Supplementary Material.
